# Healthy life expectancy for 202 countries up to 2030: Projections with a Bayesian model ensemble

**DOI:** 10.7189/jogh.13.04185

**Published:** 2023-12-27

**Authors:** Jiaxin Cai, Weiwei Hu, Yuhui Yang, Shiyu Chen, Aima Si, Yuxiang Zhang, Hui Jing, Lingmin Gong, Sitong Liu, Baibing Mi, Jiaojiao Ma, Hong Yan, Fangyao Chen

**Affiliations:** 1 Department of Epidemiology and Biostatistics, School of Public Health, Xi’an Jiaotong University, Xi’an, Shaanxi, China; 2 Department of Neurology, Xi’an Gaoxin Hospital, Xi’an, Shaanxi, China; 3 Key Laboratory for Disease Prevention and Control and Health Promotion of Shaanxi Province, Xi’an Jiaotong University, Xi’an, Shaanxi, China; 4 Department of Radiology, First Affiliate Hospital of Xi’an Jiaotong University, Xi’an, Shaanxi, China

## Abstract

**Background:**

Healthy life expectancy (HLE) projections are required for optimising social and health service management in the future. Existing studies on the topic were usually conducted by selecting a single model for analysis. We thus aimed to use an ensembled model to project the future HLE for 202 countries/region.

**Methods:**

We obtained data on age-sex-specific HLE and the sociodemographic index (SDI) level of 202 countries from 1990 to 2019 from the Global Burden of Disease (GBD) database and used a probabilistic Bayesian model comprised of 21 forecasting models to predict their HLE in 2030.

**Results:**

In general, HLE is projected to increase in all 202 countries, with the least probability of 82.4% for women and 81.0% for men. Most of the countries with the lowest projected HLE would be located in Africa. Women in Singapore have the highest projected HLE in 2030, with a 94.5% probability of higher than 75.2 years, which is the highest HLE in 2019 across countries. Maldives, Kuwait, and China are projected to have a probability of 49.3%, 41.2% and 31.6% to be the new entries of the top ten countries with the highest HLE for females compared with 2019. Men in Singapore are projected to have the highest HLE at birth in 2030, with a 93.4% probability of higher than 75.2 years. Peru and Maldives have a probability of 48.7% and 35.3% being new top ten countries in male’s HLE. The female advantage in HLE will shrink by 2030 in 117 countries, especially in most of the high SDI and European countries.

**Conclusions:**

HLE will likely continue to increase in most countries and regions worldwide in the future. More attention needs to be paid to combatting obesity, chronic diseases, and specific infectious diseases, especially in African and some Pacific Island countries. Although gender gaps may not be fully bridged, HLE could partially mitigate and even eliminate them through economic development and improvements in health care.

Improving population health encompasses not just prolonging life or increasing life expectancy at birth, but also addressing the presence of diseases and the level of functioning [1]. Significant reductions in mortality rates have been achieved in most regions of the world over the past six decades, attributed to advancements in medicine and public health, improved living standards, higher education levels, and declining fertility rates. As population aging becomes more prevalent, the importance of prioritising healthy aging is increasingly being recognized [2].

Healthy life expectancy (HLE) is a specific measurement within the broader category of population health measurements known as health expectancies [3]. Different from the naive life expectancy (LE), it integrates information on both mortality and morbidity to provide a comprehensive assessment of the health status of a population [4].

Considering the global population aging, the projection of HLE has been gaining attention, with studies consistently demonstrating its strong link to quality of life and overall health status [5]. A higher HLE among aged adults enables them to better manage diseases and health concerns while maintaining their independence [6]. Li [7] claimed that the key argument for revising the retirement/pension age should focus on HLE instead, as the accuracy in assessing the working capacity of a population significantly impacts the planning of retirement funding and health and aged care policies, and consequently, the well-being of retirees. Moreover, promoting elder quality of life and ensuring equitable distribution of limited social resources [8,9].

As for the projection of HLE, many studies have already applied various models in establishing a projection. Cao et al. [10] used multiple linear regression analysis and an autoregressive integrated moving average (ARIMA) model to analyze and project LE and HLE from 1995 to 2025. Masahiro et al. [11] developed a prediction model using an extreme gradient boosting classifier to estimate HLE without activity limitations, and subsequently applied the model to a health policy in Japan. Though these studies have contributed to the prediction of HLE, however, two research gaps remain unsolved. First, although validation demonstrated the models’ forecasting performance, it was not free of model-based uncertainty due to the modeling strategy. This means that existing HLE predictions were performed by either a single model prediction under strong theoretical assumptions, or a multi-model prediction followed by a filtering of the relatively optimal predictions, resulting in inconsistent results due to different methodologies. Specifically, this mainly relates to the likelihood and specific extent of future HLE growth being inconsistent in current research. For instance, some studies claimed that the UK HLE declined sharply in 2011 and would likely continue to do so after 2020, meaning the UK government's Aging Society Grand Challenge will be difficult to attain by 2035 [12]. Other studies indicated that the HLE of UK would increase to 70.37 in 2035 [10]. Thus, the model-based uncertainty due to model selection affects the conclusion. Second, in many studies, forecasts of HLE allow changes in future mortality rates, but assume that future health remains constant or has different deterministic scenarios. This means they do not provide any information regarding the likelihood of future health changes, presenting a significant disadvantage.

To address these issues, we employed a probabilistic Bayesian model averaging (BMA) approach for the projection of HLE, which uses an ensemble of models, each contributing probabilistically to the final projections. This enables a more comprehensive and robust assessment of HLE by considering multiple models and accounting for their respective uncertainties. Moreover, the BMA approach can generate probability intervals and probabilities to describe the uncertainty of future outcomes adequately [13].

Therefore, we applied the BMA approach to establish a model to predict the trends of HLE in 2020 and 2030 among 202 countries from three aspects: the future HLE growth, expected HLE ranking, and inequality HLE between regions and genders.

## METHODS

### Study design and aim

This is an observational study based on aggregated data obtained from the Global Burden of Disease (GBD) database. We used age- and gender-specific HLE data from 1990 to 2019 to forecast the future HLE in 2020 and 2030 using a BMA approach. Additionally, we provided the probabilities of HLE growth or decline, as well as posterior distributions of HLE for different counties and genders.

We aimed to explore three main aspects related to HLE. First, we sought to predict the probabilities and extent of future HLE increase or decrease from an overall perspective. Second, we intended to predict the expected ranking of HLE across countries, accounting for both the countries that ranking high or low in HLE and those that are projected to experience significant improvements or declines in their rankings. This ranking can inform policymakers and health care professionals in their efforts to improve population health outcomes. Third, we wanted to explore the inequality of HLE between regions and genders. By assessing disparities in health outcomes across different geographical areas and between males and females, we can identify areas where interventions are needed to promote health equity. Understanding the factors contributing to these inequalities can guide the development of targeted policies and interventions to address them effectively.

### Data sources

We collected data on age-specific male and female HLE for 1990-2019 from the GBD 2019 database for 202 countries and regions. The GBD database categorises all countries according to their SDI, which is a composite indicator that incorporates social and economic factors influencing health outcomes in each location. Based on the calculated SDI values for each year from 1990 to 2019, the 202 countries and territories were classified into five groups: low-SDI, low-middle-SDI, middle-SDI, high-middle-SDI, and high-SDI.

### BMA

Assume that *H =* {*H_1_*, *H_2_*, ..., *H_m_*} represents the set of all imputed models under consideration, and *∆* is the parameter of interest, in future forecast the posterior probability should be estimated over a period of time. Then, the posterior probability distribution of *∆* parameter for the data *D* is as follows:



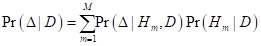



PR(*∆*|*D*) serves as the Bayesian averaging of the posterior probability ∆ under model with weighting determined by the posterior probability distribution. In the above equation, PR(*H_m_*|*D*) is the posterior distribution of the *H_m_* model:



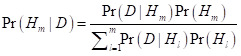



where



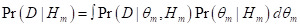



In the above equations, PR(*H_m_*|*D*) is the marginal probability of model *H_m_*, PR(*θ_m_*|*H_m_*) in the prior distribution, *θ_m_* is the vector of *H_m_* parameters and PR(*H_m_*)is the prior probability of *M* model. The BMA of this method, which is performed on all models, can provide better forecasting ability. Because



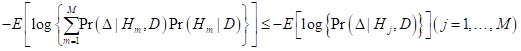



The inequality presented above demonstrates that the BMA approach is superior to the univariate model [14].

### Statistical analysis

Following the methodology described by Kontis et al. [13], we employed the BMA approach to estimate future HLE. This approach allowed for the probabilistic combination of the posterior distribution for life expectancy from multiple individual models. We included 21 individual HLE projection models *H =* {*H_1_*, *H_2_*, ..., *H_21_*} in the BMA. Importantly, this approach exhibited a smaller average projection error, thereby enhancing the validity of the projections.

These 21 models encompassed two age-time models, four weighted likelihood age-time and age-time-cohort models, eight piecewise linear age and age-time-cohort models, two age-time-cohort models, and five Lee-Carter models; they are further detailed descriptions in a previous study [13]. We assigned a weight to each model based on its projection bias so as to select the best model combination.

We employed a three-step approach to project future HLE. First, we evaluated the performance of individual models in each country, stratified by gender, withholding the data from 1990 to 2002 and using the remaining data to estimate model parameters. We calculated the projection bias using the difference between projected and withheld life expectancy. Second, we determined model weights, assigning smaller ones to models with larger projection biases. We computed the weight for each model as *exp*(–|Projection bias|) and subsequently normalised it to sum to 1. Finally, we generated projections of HLE for 2030 using all available data for each country and gender. Multiple simulations were generated from the posterior distribution of age-specific HLE under each individual model, whereby the simulations were probabilistically combined based on the model weight calculated in accordance with the aforementioned steps. Here we generated 1000 simulations from the posterior distribution, which we then aggregated to derive the posterior distribution of age-specific HLE under the BMA approach. We obtained the final estimates by taking the median of the simulations, and we calculated 95% credible intervals (95% CrI) based on the 2.5^th^ and 97.5^th^ percentiles of the simulations. We carried out all analyses in R, version 4.2.0. (R Core Team, Vienna, Austria).

## RESULTS

### Projected global HLE by age in 2020 and 2030

Our projections indicate that in 2030, male and female global HLE at birth will be 65.3 years (95% CrI = 62.5-68.0) and be 67.5 years (95% CrI = 64.8-70.3) in 2030, respectively, compared with 62.7 years (95% CrI = 61.2-64.2) and 65.1 years (95% CrI = 63.6-66.5) in 2020 (**Table 1**). Between 2020 and 2030, HLE will increase by 2.6 years (95% CrI = 1.7-6.8) for men and 2.5 years (95% CrI = 1.7-6.7) for women, while the female-male gap of HLE will be silently closing from around 2.4 years in 2020 to around 2.2 years in 2030. Moreover, our model projected the HLE at the age of 50 years between 2020 and 2030, estimating it to be 22.0 years (95% CrI = 21.3-22.7) for men and 24.2 years (95% CrI = 23.4-25.0) for women in 2020, and 23.1 years (95% CrI = 21.9-24.3) for men and 25.2 years (95% CrI = 23.9-26.5) for women in 2030. The projected difference between Health Life Expectancy (HLE) at birth and HLE at the age of 50 years is expected to increase for both men and women. For men, it is projected to increase from 40.7 years (95% CrI = 38.5-42.9) to 42.2 years (95% CrI = 38.2-46.1). For women, it is projected to increase from 40.9 years (95% CrI = 38.6-43.1) to 42.3 years (95% CrI = 38.3-46.4).

**Table 1:** The projected global HLE by age in 2020 and 2030, presented as years (predicted 95% credible intervals)

	2020		2030	
**Age in years**	**Male**	**Female**	**Male**	**Female**
0	62.7 (61.2-64.2)	65.1 (63.6-66.5)	65.3 (62.5-68.0)	67.5 (64.8-70.3)
1	63.6 (62.2-65.0)	65.8 (64.3-67.3)	65.4 (62.9-68.0)	67.6 (65.1-70.2)
5	60.4 (59.0-61.8)	62.6 (61.1-64.0)	61.8 (59.3-64.3)	64.0 (61.4-66.5)
10	55.8 (54.5-57.1)	57.9 (56.6-59.3)	57.2 (54.8-59.5)	59.3 (56.9-61.6)
15	51.2 (49.9-52.4)	53.4 (52.1-54.6)	52.5 (50.3-54.7)	54.7 (52.4-57.0)
20	46.7 (45.5-47.8)	48.9 (47.7-50.1)	48.0 (46.0-50.0)	50.2 (48.1-52.3)
25	42.3 (41.2-43.5)	44.6 (43.4-45.8)	43.6 (41.7-45.5)	45.8 (43.8-47.8)
30	38.1 (37.0-39.1)	40.3 (39.2-41.3)	39.3 (37.6-41.1)	41.5 (39.7-43.2)
35	33.9 (33.0-34.8)	36.1 (35.1-37.1)	35.1 (33.5-36.7)	37.2 (35.5-38.9)
40	29.8 (28.9-30.7)	32.0 (31.1-32.9)	31.0 (29.5-32.5)	33.1 (31.5-34.7)
45	25.8 (25.0-26.6)	28.1 (27.2-28.9)	27.0 (25.6-28.3)	29.1 (27.7-30.5)
50	22.0 (21.3-22.7)	24.2 (23.4-25.0)	23.1 (21.9-24.3)	25.2 (23.9-26.5)
55	18.4 (17.8-19.1)	20.5 (19.8-21.2)	19.4 (18.3-20.5)	21.4 (20.2-22.6)
60	15.1 (14.6-15.7)	17.0 (16.4-17.6)	16.0 (15.1-17.0)	17.8 (16.8-18.8)
65	12.1 (11.6-12.6)	13.7 (13.2-14.3)	12.9 (12.1-13.7)	14.4 (13.6-15.3)
70	9.4 (9.0-9.8)	10.8 (10.3-11.2)	10.1 (9.4-10.7)	11.4 (10.7-12.2)
75	7.1 (6.8-7.5)	8.2 (7.8-8.6)	7.7 (7.1-8.2)	8.7 (8.1-9.4)
80	5.3 (4.9-5.7)	6.1 (5.6-6.6)	5.7 (5.0-6.3)	6.5 (5.7-7.3)

### 202 countries’ projected HLE in 2020 and 2030

**Figure 1:**
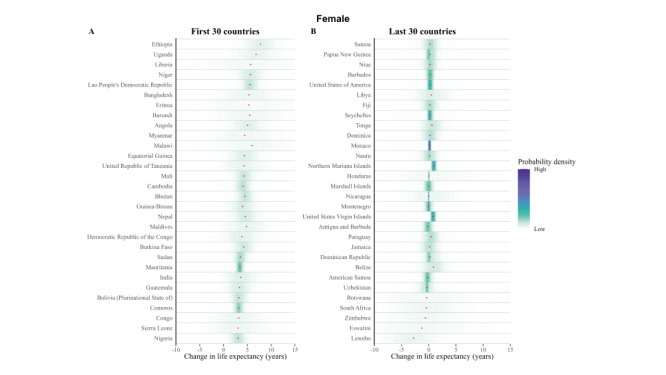
Posterior distribution of projected change in female HLE at birth from 2020 to 2030.

According to the projections for 2020, male HLE at birth varied greatly across countries, ranging from 42.6 years (95% CrI = 41.1-44.1) in Lesotho to 74.1 years (95% CrI = 72.4-75.8) in Singapore. Similarly, female HLE at birth ranges from 46.7 years (95% CrI = 44.9-48.6) in Lesotho to 75.6 years (95% CrI = 73.9-77.2) in Singapore. Looking ahead to 2030, the projections of male HLE at birth ranged from 40.4 years (95% CrI = 37.3-43.5) in Lesotho to 77.4 years (95% CrI = 74.3-80.5) in Singapore. Notably, the female HLE at birth is projected to improve,with estimations suggesting it will range from 44.1 years (95% CrI = 40.0-48.2) in Lesotho to 78.2 years (95% CrI = 75.2-81.2) in Singapore (**Figure 1** and Table S1 in the **Online Supplementary Document**). According to our projections, Lesotho's HLE is expected to remain at the lowest level, with a forecasted decrease in male HLE. Meanwhile, the model projected an increased gap in HLE at birth between local areas with the highest and lowest levels, with increases of 5.5 years (95% CrI = 4.1-7.1) for men and 5.2 years (95% CrI = 3.9-7.5) for women. Our model also projected a widening gap in HLE at birth between local areas with the highest and lowest levels, with an expected increase by 5.5 years (95% CrI = 4.1-7.1 for men and 5.2 years (95% CrI = 3.9-7.5) for women.

### Posterior distribution of projected change in life expectancy at birth from 2020 to 2030

**Figure 2:**
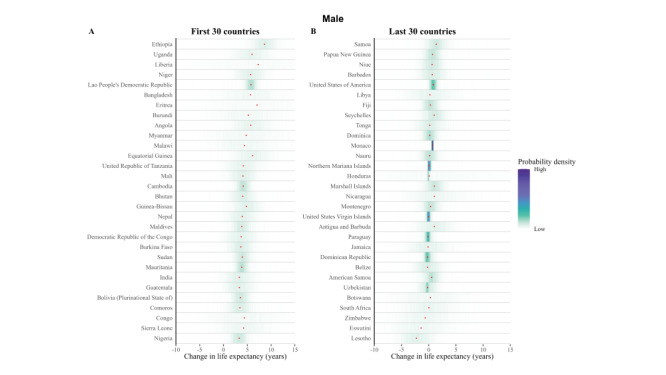
Posterior distribution of projected change in male HLE at birth from 2020 to 2030.

Taking model uncertainty into account, we project that the HLE will increase in all 202 countries with a least probability of 82.4% for women and 81.0% for men, although the increase varies across countries (**Figure 1** and **Figure 2**).

### Posterior distribution of projected male’s and female’s HLE at birth from 2020 to 2030

**Figure 3:**
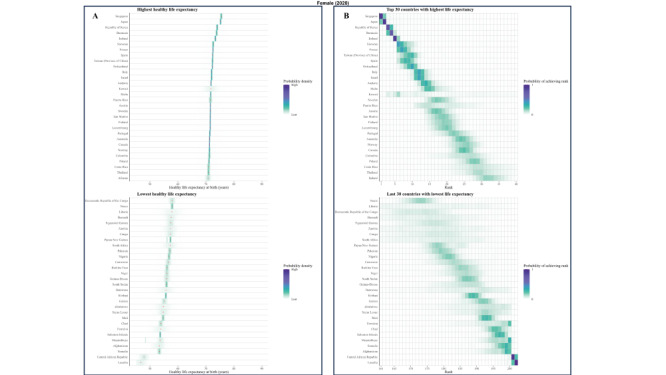
Projected female’s HLE at birth in 2020 **Panel A.** Posterior distribution of female’s HLE and its median value. Red dots show the posterior medians. **Panel B.** Probability distribution for the country’s rank.

**Figure 4:**
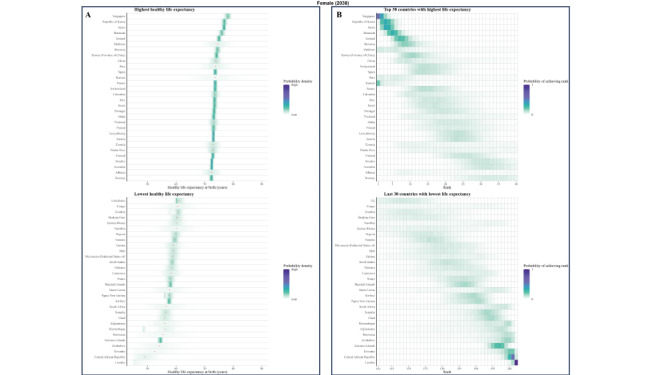
Projected female’s HLE at birth in 2030. **Panel A.** Posterior distribution of female’s HLE and its median value. Red dots show the posterior medians. **Panel B.** Probability distribution for the country’s rank.

**Figure 5:**
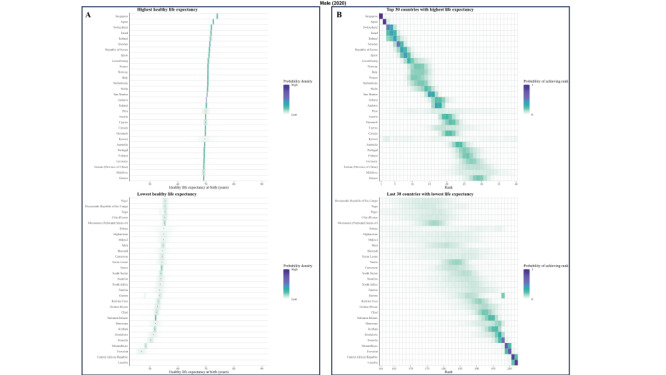
Projected male’s HLE at birth in 2020. **Panel A.** Posterior distribution of male’s HLE and its median value. Red dots show the posterior medians. **Panel B.** Probability distribution for the country’s rank.

**Figure 6:**
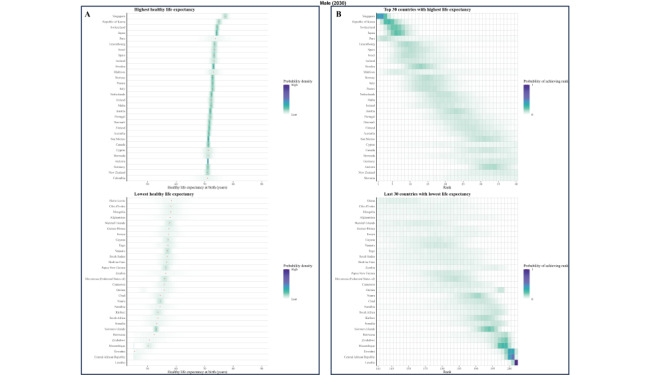
Projected male’s HLE at birth in 2030. **Panel A.** Posterior distribution of male’s HLE and its median value. Red dots show the posterior medians. **Panel B.** Probability distribution for the country’s rank.

We explored the frequency distributions of projected HLE at birth for males and females across various countries, along with the corresponding rank distribution for the first 30 and last 30 nations by rank (**Figure 3**, **Figure 4**, **Figure 5**, and **Figure 6**). There is a 94.5% probability that HLE at birth for Singapore women and 93.4% for Singapore men will be higher than 75.2 years in 2030, the same as the highest life expectancy in the world in 2019. The probability that Singapore women will have the highest life expectancy in the world in 2030 is 40.3%, with a 29.4% probability of being in second place. As for Singapore male, the probability is expected 28.7% and 31.3% for these two proportions.

According to our projections, eight of the top ten countries with the highest male HLE in 2030 are currently in the top ten in 2019, except for Peru and Maldives as new entrants into this group. Similarly, we projected that seven of the top ten countries with the highest female HLEs in 2019 will also be in the the top ten for 2030, i.e. all except for Maldives, Kuwait, and China. Female HLE in Singapore is followed by those in Republic of Korea and Japan, the projected distributions of which overlap substantially. For example, although median projected HLE for Japan places it in third place in 2030, there is a 14.6% probability that Japanese women will continue to have the first or second highest highest female life expectancy in 2030.

Maldives, Kuwait, and China are also projected to have large gains in female longevity, with their female HLEs having probabilities of 49.3%, 41.2% and 31.6% to be among the top of all countries in 2030, which is a substantial improvement to their rankings in 2019. For men, Singapore is still expected to rank first, followed by the Republic of Korea and Switzerland. Peru and Maldives are anticipated to make considerable progress in male longevity, with a substantial likelihood of 48.7% and 35.3%, respectively, for being among the top ten countries by HLE in 2030. This likewise presents a significant advancement in their rankings compared with 2019. As a result of these trends, by 2030, Republic of Korea is likely to take Japan’s rank for HLE for women and men, taking the second position. The Maldives are projected to make large gains for both genders.

As for some countries, their HLE is not very optimistic. In 2020, the proportion of Africa countries in ten countries with the lowest projected HLE is seven of ten for male and eight of ten for female. In 2030, seven out of ten countries with the lowest projected male HLE will likely be located in Africa, particularly in the southern and eastern regions such as Lesotho, Eswatini, Mozambique, and others. Similarly, for females, seven out of ten countries will be situated in Africa. The HLE of Solomon Islands and Afghanistan are also not projected to be favorable.

### Men’s and Women’s HLE at age 50 years in 2020 and 2030

In 2020, HLE at age 50 years was highest in Japanese women, at 34.5 years (95% CrI = 34.4-34.6), followed by Singaporean and Bermuda women at 34.3 years (95% CrI = 34.1-34.4) and 33.4 years (95% CrI = 33.3-33.6), respectively. By 2030, it is projected that in 57 of the countries analysed, women aged 50 years will have a greater than 50% probability of having a HLE exceeding 30 years. Meanwhile, men aged 50 years will have above 50% probability of surpassing a HLE of 30 years in 32 countries. Among the 202 countries, the bottom ten in terms of female life expectancy are primarily comprised of small island nations in Oceania, and some countries in the eastern and southern regions of Africa. A similar pattern is observed for the bottom ten in male life expectancy, with Africa dominating.

### The gap of HLE between men and women

**Figure 7:**
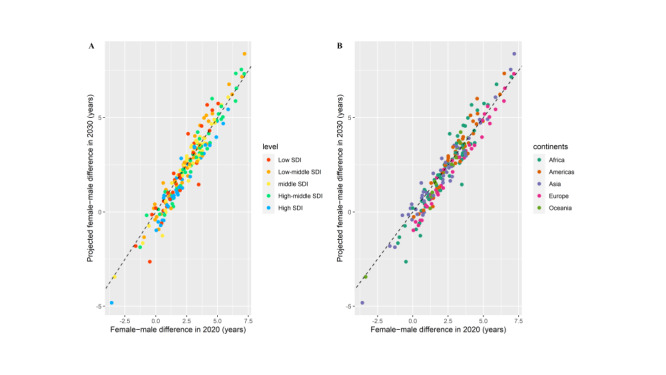
HLE difference between female and male in 2030 vs 2020. The points show the difference of posterior median HLE at birth between female and male in 2030 and 2020. The color of each point denotes the country’s SDI level and geographical region. **Panel A.** Country’s SDI level. **Panel B.** Geographical region.

In 2020, women had a higher life expectancy than men by -3.6 (Qatar) to 7.2 (Mongolia) years (**Figure 7**). We project that the female advantage will shrink by 2030 in 117 countries, especially in most high SDI countries and most European countries, where a slightly larger HLE gain is projected for women than for men, while in 71 countries the differences will increase. For countries in Oceania, the HLE appears to have fewer differences than other countries. The advantage of women is not universal across all countries, approximately 11 countries are expected to exhibit such an advantage, with the majority of these countries being in Asia and Africa, both in 2020 and 2030.

## DISCUSSION

Here we used a BMA approach for modeling HLE to forecast 202 countries’ HLE in 2020 and 2030. We also generated probability intervals and probabilities to describe the uncertainty of future outcomes adequately [15]. This study, to our knowledge, is the most comprehensive assessment of trends in HLE across countries and over time, covering 202 countries from 1990 to 2030.

Our study had three main findings. First, our results indicate that there is a high likelihood (exceeding 80%) that HLE will continue to increase in all 202 countries. Second, there are still significant disparities in HLE among different countries, with some countries leading (e.g. Singapore) in this aspect and others lagging (e.g. Lesotho and some Africa countries and Pacific Island countries). This highlights the need for targeted interventions and policies to address the underlying factors contributing to these disparities and promote equitable access to health care and resources globally. Third, the gender gap in HLE has shown a slight decrease across countries, especially in high SDI countries and European nations. This indicates progress in addressing gender-based health inequalities, but further efforts are still needed to achieve true gender equity in health outcomes worldwide.

Regarding the estimated growth in HLE, the increase is projected to be largest in Ethiopia, Liberia, and Eritrea for men and in Ethiopia, Uganda, and Malawi for women. We noticed that these countries are most located in Africa, especially in northeast of Africa. However, this does not necessarily imply that the future HLE in these countries will reach a significantly high level. Based on our projections for 2030, these countries are still expected to face challenges in achieving a satisfactory HLE, although their situation is relatively better compared to countries in southern Africa.

While for those industrialized/developed countries such as USA, Canada, Japan, Germany, France, United Kingdom, and Australia, the increase in HLE is not as substantial, mostly falling in the intermediate or lower range, for both genders. Moreover, the HLE of USA is not optimistic. The reasons for improvement of HLE of USA falling behind may include the absence of universal health insurance for all residents, drug abuse, a high child mortality rate, and a significant increase in obesity levels, and the increasing incidence rate of chronic [16-19]. The prevalence of chronic diseases has given rise to heightened health care needs, necessitating enhanced investments in health care expenditure and optimised use of health care infrastructure. Insufficient allocation of resources and suboptimal usage of health care facilities persist as significant obstacles in the pursuit of improved population health globally. Addressing these challenges is imperative to foster favorable health outcomes and well-being for individuals worldwide [20].

Furthermore, we noticed that the projected gains (increases) in HLE are anticipated to be concentrated primarily among older age groups (50 years old and above groups), with women experiencing a more pronounced effect. This is expected to contribute to the ongoing demographic shift toward an aging population in industrialized nations. Kontis et al. [13] found a similar pattern but about the life expectancy. We assume this pattern is the result of two phenomena. First, younger generations are under increasing pressure (from life or work), and the onset of certain chronic diseases among the elderly is gradually occurring at a younger age [21-23]. Second, the economic conditions of the 50-year-old and above population are favorable comparing with the youngers, allowing them to access better health care services. Meanwhile, the competition in the workplace has mostly plateaud, and the focus is shifting back to personal life. Although the current level of HLE at 50 years old is considerable, excessive consumption during youth may potentially diminish HLE in later older populations. It remains uncertain whether the HLE in the future for the older age groups will be able to maintain the current level. Therefore, attention should be given to the living conditions, stress levels, and health care of young individuals, promoting healthy lifestyles, enhancing health education, and implementing corresponding policies to alleviate the pressures faced by young people.

Second, in terms of the prediction ranking of HLE in 2020 and 2030, according to our projection, we found Singapore will likely have the highest HLE for both genders in 2030. South Korea is expected to be in a relatively optimistic position due to having one of the lowest obesity rates globally, along with good nutrition, a lower number of smokers, and excellent access to health care [24]. Meanwhile, excess BMI, especially among urban women, increased in sub-Saharan Africa countries from 2.59 kg/m^-2^ (95% CrI = 2.21-2.98) in 1985 to 3.17 kg/m^-2^ (95% CrI = 2.93-3.42) in 2017 [25], which may result in southern African countries having low HLE. Moreover, many of the Pacific Island countries have the highest obesity rates, with as many as four out of five individuals being obese or overweight [26]; the HLE of these countries is likewise low.

We have also taken note of several countries that are expected to enter the top ten rankings in terms of male and female HLE. Maldives is expected to make great gains in both male and female. Over the course of time, Maldives has achieved notable advancements, with a health care expenditure exceeding 9% of its gross domestic product [27]. This substantial investment demonstrates a strong dedication toward attaining universal health coverage and meeting the targets outlined in the Sustainable Development Goals. Moreover, Maldives has achieved remarkable success in addressing various diseases that are prevalent globally [28].

The countries in the southern and eastern regions of Africa, such as Lesotho, Eswatini, etc., and those in Oceania, including the Solomon Islands and others, will continue to be markedly disadvantaged, which is in line with the projections made by Foreman et la. [29] in 2018 on these countries’ life expectancies. As for Lesotho, previous studies find that the HLE of Lesotho was at 44.2 years in 2019, presenting a decrease from 46.6 years in 2016, i.e. a change of 5.03% [30]. Our projection showed similar results. This can be due to two reasons, the first being that Lesotho has a generalised human immunodeficiency virus (HIV) epidemic. In 2017, the overall prevalence of HIV among individuals aged 15-59 years was estimated to be 25.6% (95% CrI = 24.7-26.4), which corresponds to approximately 306 000 people living with HIV [31]. The second reason can be attributed to poverty, limited social protection measures, and inadequate health care resources [32]. Lesotho is facing the challenge of addressing the needs and rights of its rapidly growing ageing population. To enhance the country’s life expectancy, we advise that policymakers prioritise resource allocation toward the modifiable risk factors that contribute to significant premature mortality. By emphasising the management and prevention of these modifiable risks, there is the potential to enhance HLE in the long term. Some studies claim that during 1990 and 2019, the four countries at a disadvantage that have experienced declines in HLE at birth are Lesotho, Eswatini, Zimbabwe, and Uzbekistan, which is similar with our projection [30].

Third, we not only focused on the inequality between countries, but also conducted in-depth research on gender inequality. The gender gap in HLE has slightly decreased across countries, particularly in some high SDI countries and European nations (**Figure 7**). Among the countries projected to have the HLE for men exceeding 70 years in 2030, approximately 83.3% of them have a HLE gap of less than two years. For other countries, this proportion is approximately 63.8%. Consequently, there could be a positive correlation between gender equality and HLE in men and women, with higher the gender equality correlating with higher HLE in both genders. Therefore, we speculate that although life expectancy may not completely eliminate gender gap, there is a possibility that HLE can partially mitigate these gaps to some extent, and even eliminate them entirely, with the economic development and the improvement health care conditions, and so on.

One of the significant strengths of our study lies in the use of the BMA approach to integrate projections from multiple models, thereby comprehensively capturing the uncertainty surrounding future trends in HLE. The effectiveness of this approach in accounting for model choice uncertainty has been validated previously, and it has demonstrated a smaller average projection error compared to the best individual model [33].

There still some limitation in this study. First, while summarised data offers a convenient way to analyze large data sets, we may have failed to capture the intricacies of individual cases, leading to biased predictions. Second, the effectiveness of the BMA approach is influenced by prior distribution, which represent initial assumptions about parameters before data observation. Priors can improve accuracy by incorporating existing knowledge but may introduce bias or subjectivity.

## CONCLUSIONS

Our projections indicate a high probability (≥80%) of continued increases in HLE across 202 countries. However, to ensure sustained progress, greater efforts are needed to address the prevalence of obesity, chronic diseases, and specific infectious diseases, particularly in African and some Pacific Island countries. While life expectancy alone may not completely eliminate gender disparities, we speculate that improvements in economic development and health care conditions have the potential to partially mitigate and potentially eliminate these gaps in HLE.

## Additional material


Online Supplementary Document

## References

[1] Crimmins EM Lifespan and Healthspan: Past, Present, and Promise. Gerontologist. 2015;55:901-11. 10.1093/geront/gnv130 26561272 PMC4861644

[2] Salomon JA Wang H Freeman MK Vos T Flaxman AD Lopez AD Healthy life expectancy for 187 countries, 1990–2010: a systematic analysis for the Global Burden Disease Study 2010. Lancet. 2012;380:2144-62. 10.1016/S0140-6736(12)61690-0 23245606

[3] Stiefel MC Perla RJ Zell BL A Healthy Bottom Line: Healthy Life Expectancy as an Outcome Measure for Health Improvement Efforts. Milbank Q. 2010;88:30-53. 10.1111/j.1468-0009.2010.00588.x 20377757 PMC2888015

[4] Saito Y Robine JM Crimmins EM The methods and materials of health expectancy. Stat J IAOS. 2014;30:209-23. 30319718 10.3233/SJI-140840 PMC6178833

[5] Martinez R Morsch P Soliz P Hommes C Ordunez P Vega E Life expectancy, healthy life expectancy, and burden of disease in older people in the Americas, 1990–2019: a population-based study. Rev Panam Salud Publica. 2021;45:e114. 10.26633/RPSP.2021.114 34621302 PMC8489742

[6] Rechel B Grundy E Robine JM Cylus J Mackenbach JP Knai C Ageing in the European Union. Lancet. 2013;381:1312-22. 10.1016/S0140-6736(12)62087-X 23541057

[7] Li J Bayesian joint modelling of life expectancy and healthy life expectancy and valuation of retirement village contract. Scand Actuar J. 2023:1-19. 10.1080/03461238.2023.2232816

[8] Stewart ST Cutler DM Rosen AB US Trends in Quality-Adjusted Life Expectancy From 1987 to 2008: Combining National Surveys to More Broadly Track the Health of the Nation. Am J Public Health. 2013;103:e78-87. 10.2105/AJPH.2013.301250 24028235 PMC3828687

[9] Crimmins EM Hayward MD Hagedorn A Saito Y Brouard N Change in disability-free life expectancy for Americans 70 years old and older*. Demography. 2009;46:627-46. 10.1353/dem.0.0070 19771948 PMC2831348

[10] Cao X Hou Y Zhang X Xu C Jia P Sun X A comparative, correlate analysis and projection of global and regional life expectancy, healthy life expectancy, and their GAP: 1995-2025. J Glob Health. 2020;10:020407. 10.7189/jogh.10.020407 33110572 PMC7568920

[11] Nishi M Nagamitsu R Matoba S Development of a Prediction Model for Healthy Life Years Without Activity Limitation: National Cross-sectional Study. JMIR Public Health Surveill. 2023;9:e46634. 10.2196/46634 37195737 PMC10233441

[12] Welsh CE Matthews FE Jagger C Trends in life expectancy and healthy life years at birth and age 65 in the UK, 2008-2016, and other countries of the EU28: An observational cross-sectional study. Lancet Reg Health Eur. 2021;2:100023. 10.1016/j.lanepe.2020.100023 33870247 PMC8042672

[13] Kontis V Bennett JE Mathers CD Li G Foreman K Ezzati M Future life expectancy in 35 industrialised countries: projections with a Bayesian model ensemble. Lancet. 2017;389:1323-35. 10.1016/S0140-6736(16)32381-9 28236464 PMC5387671

[14] Bahrami S Hajian-Tilaki K Bayani M Chehrazi M Mohamadi-Pirouz Z Amoozadeh A Bayesian model averaging for predicting factors associated with length of COVID-19 hospitalization. BMC Med Res Methodol. 2023;23:163. 10.1186/s12874-023-01981-x 37415112 PMC10326965

[15] Jagger C Gillies C Moscone F Cambois E Van Oyen H Nusselder W Inequalities in healthy life years in the 25 countries of the European Union in 2005: a cross-national meta-regression analysis. Lancet. 2008;372:2124-31. 10.1016/S0140-6736(08)61594-9 19010526

[16] Singh GK Yu SM Infant Mortality in the United States, 1915-2017: Large Social Inequalities have Persisted for Over a Century. Int J MCH AIDS. 2019;8:19-31. 10.21106/ijma.271 31049261 PMC6487507

[17] Anderson E Durstine JL Physical activity, exercise, and chronic diseases: A brief review. Sports Med Health Sci. 2019;1:3-10. 10.1016/j.smhs.2019.08.006 35782456 PMC9219321

[18] Kadri AN Wilner B Hernandez AV Nakhoul G Chahine J Griffin B Geographic Trends, Patient Characteristics, and Outcomes of Infective Endocarditis Associated With Drug Abuse in the United States From 2002 to 2016. J Am Heart Assoc. 2019;8:e012969. 10.1161/JAHA.119.012969 31530066 PMC6806029

[19] Douthit N Kiv S Dwolatzky T Biswas S Exposing some important barriers to health care access in the rural USA. Public Health. 2015;129:611-20. 10.1016/j.puhe.2015.04.001 26025176

[20] Mackenbach JP Karanikolos M McKee M The unequal health of Europeans: successes and failures of policies. Lancet. 2013;381:1125-34. 10.1016/S0140-6736(12)62082-0 23541053

[21] Rossi RC Vanderlei LCM Gonçalves ACCR Vanderlei FM Bernardo AFB Yamada KMH Impact of obesity on autonomic modulation, heart rate and blood pressure in obese young people. Auton Neurosci. 2015;193:138-41. 10.1016/j.autneu.2015.07.424 26260435

[22] Hinton TC Adams ZH Baker RP Hope KA Paton JFR Hart EC Investigation and Treatment of High Blood Pressure in Young People. Hypertension. 2020;75:16-22. 10.1161/HYPERTENSIONAHA.119.13820 31735086

[23] Williams B High blood pressure in young people and premature death. BMJ. 2011;342:d1104. 10.1136/bmj.d1104 21343188

[24] Di Cesare M Sorić M Bovet P Miranda JJ Bhutta Z The epidemiological burden of obesity in childhood: a worldwide epidemic requiring urgent action. BMC Med. 2019;17:212. 10.1186/s12916-019-1449-8 31760948 PMC6876113

[25] NCD Risk Factor Collaboration (NCD-RisC) Rising rural body-mass index is the main driver of the global obesity epidemic in adults. Nature. 2019;569:260-4. 10.1038/s41586-019-1171-x 31068725 PMC6784868

[26] Tong TJ Mohammadnezhad M Alqahtani NS Determinants of overweight and obesity and preventive strategies in Pacific countries: a systematic review. Glob Health J. 2022;6:122-8. 10.1016/j.glohj.2022.07.005

[27] Wang H Torres LV Travis P Financial protection analysis in eight countries in the WHO South-East Asia Region. Bull World Health Organ. 2018;96:610-620E. 10.2471/BLT.18.209858 30262942 PMC6154066

[28] Banks LM O’Fallon T Hameed S Usman SK Polack S Kuper H Disability and the achievement of Universal Health Coverage in the Maldives. PLoS One. 2022;17:e0278292. 10.1371/journal.pone.0278292 36542614 PMC9770361

[29] Foreman KJ Marquez N Dolgert A Fukutaki K Fullman N McGaughey M Forecasting life expectancy, years of life lost, and all-cause and cause-specific mortality for 250 causes of death: reference and alternative scenarios for 2016–40 for 195 countries and territories. Lancet. 2018;392:2052-90. 10.1016/S0140-6736(18)31694-5 30340847 PMC6227505

[30] GBD 2019 Demographics Collaborators Global age-sex-specific fertility, mortality, healthy life expectancy (HALE), and population estimates in 204 countries and territories, 1950–2019: a comprehensive demographic analysis for the Global Burden of Disease Study 2019. Lancet. 2020;396:1160-203. 10.1016/S0140-6736(20)30977-6 33069325 PMC7566045

[31] Schwitters A McCracken S Frederix K Tierney R Koto M Ahmed N High HIV prevalence and associated factors in Lesotho: Results from a population-based survey. PLoS One. 2022;17:e0271431. 10.1371/journal.pone.0271431 35901094 PMC9333200

[32] Slater R Cash transfers, social protection and poverty reduction. Int J Soc Welf. 2011;20:250-9. 10.1111/j.1468-2397.2011.00801.x

[33] Bai R Liu Y Zhang L Dong W Bai Z Zhou M Projections of future life expectancy in China up to 2035: a modelling study. Lancet Public Health. 2023;8:e915-e922. 10.1016/S2468-2667(22)00338-3 37004714 PMC10188127

