# Supplementary material for: Healthy life expectancy for 202 countries up to 2030: Projections with a Bayesian model ensemble
Source: J Glob Health. 2023 Dec 27;13:04185. doi: 10.7189/jogh.13.04185 (PMC10750449; doi:10.7189/jogh.13.04185)
Supplement: Online Supplementary Document [file jogh-13-04185-s001.pdf]

Supplementary tables

Table S1. The projected HLE at birth and corresponding 95% CI across 202 countries in 2020 and 2030

	2020		2030	
Location	Male	Female	Male	Female
Afghanistan	55.0 (53.2 - 56.8)	53.4 (51.6 - 55.3)	58.4 (54.1 - 62.6)	56.4 (52.3 - 60.5)
Albania	67.2 (65.5 - 69.0)	70.9 (69.3 - 72.6)	69.3 (65.8 - 72.8)	72.5 (69.3 - 75.7)
Algeria	67.1 (65.4 - 68.7)	66.0 (64.4 - 67.5)	69.9 (66.9 - 73.0)	68.2 (65.4 - 71.1)
American Samoa	61.7 (60.2 - 63.2)	63.5 (62.1 - 65.0)	62.2 (59.5 - 64.9)	63.4 (60.8 - 66.0)
Andorra	70.2 (68.7 - 71.8)	71.8 (70.2 - 73.5)	71.2 (68.5 - 74.0)	72.4 (69.4 - 75.3)
Angola	56.0 (54.4 - 57.6)	58.7 (57.1 - 60.3)	61.8 (58.2 - 65.5)	63.8 (60.4 - 67.3)
Antigua and Barbuda	65.9 (64.2 - 67.5)	67.3 (65.7 - 68.9)	66.9 (63.6 - 70.3)	67.2 (64.5 - 70.0)
Argentina	65.3 (63.8 - 66.7)	68.5 (66.9 - 70.0)	66.6 (63.8 - 69.3)	69.4 (66.7 - 72.2)
Armenia	65.1 (63.4 - 66.7)	68.6 (67.1 - 70.2)	66.9 (63.4 - 70.3)	70.1 (67.0 - 73.1)
Australia	69.7 (68.1 - 71.2)	71.3 (69.7 - 73.0)	71.6 (68.7 - 74.4)	72.5 (69.6 - 75.4)
Austria	70.0 (68.4 - 71.5)	71.7 (70.1 - 73.3)	72.1 (69.2 - 75.0)	73.1 (70.3 - 76.0)
Azerbaijan	61.5 (59.8 - 63.2)	64.1 (62.4 - 65.8)	63.5 (60.1 - 67.0)	65.1 (62.0 - 68.2)
Bahamas	62.3 (60.8 - 63.8)	66.5 (64.9 - 68.1)	63.1 (60.3 - 65.9)	67.2 (64.2 - 70.1)
Bahrain	67.3 (65.5 - 69.0)	65.9 (64.2 - 67.6)	69.6 (65.8 - 73.4)	67.6 (64.3 - 70.8)
Bangladesh	65.2 (63.2 - 67.2)	65.1 (63.3 - 67.0)	71.1 (65.9 - 76.2)	70.6 (66.2 - 75.0)
Barbados	66.0 (64.4 - 67.7)	67.2 (65.7 - 68.7)	66.8 (63.6 - 70.0)	67.5 (64.8 - 70.2)
Belarus	61.5 (59.8 - 63.2)	68.6 (67.0 - 70.2)	62.3 (58.7 - 66.0)	69.6 (66.5 - 72.6)
Belgium	69.1 (67.5 - 70.7)	70.5 (69.0 - 72.1)	70.8 (67.9 - 73.7)	71.5 (68.7 - 74.3)
Belize	63.0 (61.4 - 64.7)	67.1 (65.5 - 68.7)	62.9 (59.7 - 66.1)	68.0 (65.0 - 71.0)
Benin	55.9 (54.4 - 57.4)	58.2 (56.8 - 59.7)	58.9 (56.2 - 61.7)	61.3 (58.6 - 64.0)
Bermuda	68.9 (67.3 - 70.6)	73.9 (72.2 - 75.6)	71.3 (68.2 - 74.3)	76.2 (73.1 - 79.2)
Bhutan	63.8 (62.1 - 65.4)	63.7 (62.0 - 65.4)	67.9 (64.5 - 71.2)	68.3 (65.2 - 71.4)
Bolivia (Plurinational State of)	63.4 (61.7 - 65.1)	63.4 (61.8 - 65.0)	67.0 (63.8 - 70.2)	66.8 (63.7 - 69.8)
Bosnia and Herzegovina	65.9 (63.5 - 68.3)	68.6 (66.7 - 70.6)	67.7 (60.9 - 74.4)	69.7 (65.2 - 74.3)
Botswana	52.1 (50.1 - 54.1)	55.8 (53.5 - 58.1)	52.5 (47.1 - 58.0)	55.8 (49.6 - 61.9)
Brazil	63.5 (61.9 - 65.1)	67.5 (66.0 - 69.1)	66.3 (63.5 - 69.2)	70.1 (67.3 - 73.0)
Brunei Darussalam	64.3 (62.7 - 65.9)	66.4 (64.9 - 68.0)	66.0 (62.9 - 69.1)	67.7 (65.0 - 70.4)
Bulgaria	62.3 (60.8 - 63.8)	67.2 (65.7 - 68.8)	63.4 (60.6 - 66.1)	68.3 (65.5 - 71.1)
Burkina Faso	53.1 (51.6 - 54.5)	56.2 (54.8 - 57.7)	56.8 (54.0 - 59.7)	60.6 (57.7 - 63.4)
Burundi	54.5 (52.5 - 56.6)	57.7 (55.8 - 59.6)	60.0 (54.1 - 65.9)	63.5 (58.2 - 68.8)
Cabo Verde	61.6 (59.3 - 63.9)	67.4 (65.8 - 69.0)	62.2 (57.7 - 66.8)	68.9 (65.7 - 72.1)
Cambodia	59.9 (58.4 - 61.4)	63.1 (61.5 - 64.7)	64.1 (61.2 - 67.0)	67.3 (64.2 - 70.4)
Cameroon	54.2 (52.7 - 55.7)	56.4 (54.9 - 58.0)	55.8 (52.6 - 58.9)	58.2 (54.9 - 61.6)
Canada	70.0 (68.3 - 71.6)	71.4 (69.8 - 72.9)	71.5 (68.5 - 74.4)	72.1 (69.3 - 74.9)
Central African Republic	43.4 (42.1 - 44.7)	47.8 (46.2 - 49.4)	44.8 (41.9 - 47.7)	49.3 (45.6 - 52.9)
Chad	52.4 (50.9 - 53.8)	53.9 (52.4 - 55.4)	54.5 (51.5 - 57.6)	56.2 (53.2 - 59.1)
Chile	68.5 (67.0 - 70.0)	70.3 (68.6 - 71.9)	70.6 (67.7 - 73.5)	72.0 (69.0 - 74.9)
China	67.6 (66.0 - 69.3)	70.6 (68.9 - 72.3)	70.4 (67.4 - 73.4)	74.0 (70.9 - 77.1)
Colombia	68.3 (66.6 - 70.0)	71.4 (69.7 - 73.0)	71.4 (67.6 - 75.1)	73.9 (70.8 - 77.0)
Comoros	60.0 (58.4 - 61.5)	61.1 (59.6 - 62.6)	63.5 (60.4 - 66.6)	64.4 (61.7 - 67.2)

Congo	57.2 (55.2 - 59.1)	57.4 (55.7 - 59.1)	61.7 (56.3 - 67.2)	60.7 (56.5 - 64.9)
Cook Islands	63.9 (62.4 - 65.4)	67.3 (65.7 - 69.0)	65.3 (62.3 - 68.3)	69.1 (65.8 - 72.4)
Costa Rica	67.8 (66.2 - 69.4)	71.1 (69.5 - 72.7)	68.3 (65.3 - 71.3)	72.1 (69.2 - 75.1)
Côte d'Ivoire	55.2 (53.6 - 56.8)	58.0 (56.3 - 59.6)	58.4 (54.8 - 62.0)	61.1 (57.1 - 65.0)
Croatia	66.6 (65.0 - 68.2)	70.3 (68.7 - 71.9)	68.8 (65.7 - 71.9)	71.9 (68.9 - 74.8)
Cuba	67.2 (65.6 - 68.8)	69.8 (68.2 - 71.4)	68.0 (65.0 - 71.0)	71.0 (68.0 - 74.0)
Cyprus	69.9 (68.2 - 71.6)	70.6 (69.0 - 72.2)	71.3 (68.4 - 74.3)	72.2 (69.3 - 75.1)
Czechia	67.0 (65.4 - 68.6)	70.6 (69.0 - 72.3)	69.5 (66.6 - 72.4)	72.4 (69.5 - 75.3)
Democratic People's Republic of Korea	63.5 (60.8 - 66.1)	66.8 (64.3 - 69.2)	65.3 (57.3 - 73.3)	68.7 (61.7 - 75.7)
Democratic Republic of the Congo	55.4 (53.9 - 56.9)	57.8 (56.3 - 59.4)	59.2 (55.9 - 62.5)	61.8 (58.4 - 65.1)
Denmark	69.9 (68.3 - 71.5)	70.5 (68.9 - 72.1)	71.9 (68.9 - 74.9)	72.0 (69.1 - 74.8)
Djibouti	58.4 (56.8 - 59.9)	60.0 (58.5 - 61.6)	60.6 (57.4 - 63.8)	61.9 (58.9 - 64.8)
Dominica	61.8 (60.3 - 63.3)	64.9 (63.3 - 66.4)	62.0 (59.3 - 64.7)	65.1 (62.4 - 67.9)
Dominican Republic	61.9 (60.5 - 63.4)	66.3 (64.7 - 67.8)	61.8 (59.3 - 64.4)	66.8 (63.9 - 69.6)
Ecuador	65.4 (63.6 - 67.1)	67.7 (65.9 - 69.4)	66.4 (63.1 - 69.7)	68.9 (65.6 - 72.2)
Egypt	62.6 (60.9 - 64.4)	62.2 (60.4 - 63.9)	64.7 (60.7 - 68.7)	64.1 (60.3 - 67.9)
El Salvador	62.2 (60.3 - 64.1)	68.4 (66.6 - 70.3)	63.8 (59.4 - 68.3)	70.2 (66.4 - 74.0)
Equatorial Guinea	57.1 (55.3 - 58.9)	57.6 (56.0 - 59.2)	63.3 (59.3 - 67.3)	62.1 (58.9 - 65.2)
Eritrea	55.0 (52.3 - 57.7)	58.5 (56.1 - 60.9)	62.7 (53.6 - 71.8)	64.3 (56.9 - 71.7)
Estonia	65.9 (64.2 - 67.7)	70.9 (69.3 - 72.5)	69.3 (65.4 - 73.2)	73.1 (69.9 - 76.3)
Eswatini	47.0 (45.1 - 48.9)	54.0 (51.6 - 56.3)	45.9 (41.1 - 50.7)	53.1 (46.6 - 59.6)
Ethiopia	60.0 (58.4 - 61.6)	61.9 (60.3 - 63.6)	68.7 (65.1 - 72.3)	69.8 (66.1 - 73.6)
Fiji	58.6 (57.1 - 60.0)	60.7 (59.2 - 62.1)	59.0 (56.3 - 61.7)	61.0 (58.4 - 63.6)
Finland	69.5 (67.9 - 71.1)	71.6 (70.0 - 73.2)	71.7 (68.9 - 74.5)	73.0 (70.2 - 75.8)
France	70.8 (69.2 - 72.4)	72.6 (70.9 - 74.2)	73.0 (70.0 - 76.0)	73.9 (71.0 - 76.8)
Gabon	57.2 (55.8 - 58.7)	60.7 (59.1 - 62.4)	59.8 (57.1 - 62.5)	62.6 (59.3 - 66.0)
Gambia	57.9 (56.5 - 59.4)	58.8 (57.2 - 60.4)	60.0 (57.3 - 62.8)	60.3 (57.4 - 63.2)
Georgia	60.9 (59.2 - 62.5)	67.7 (66.1 - 69.4)	61.8 (58.4 - 65.1)	69.2 (65.8 - 72.6)
Germany	69.3 (67.7 - 71.0)	70.4 (68.8 - 72.0)	71.2 (68.3 - 74.1)	71.5 (68.6 - 74.3)
Ghana	56.4 (54.7 - 58.0)	59.9 (58.4 - 61.4)	58.1 (55.1 - 61.0)	62.5 (59.7 - 65.3)
Greece	69.3 (67.7 - 70.8)	70.8 (69.2 - 72.4)	70.2 (67.5 - 72.9)	71.8 (68.8 - 74.7)
Greenland	62.3 (60.8 - 63.8)	64.4 (62.7 - 66.0)	64.8 (62.1 - 67.5)	66.8 (63.5 - 70.1)
Grenada	62.7 (61.1 - 64.2)	65.4 (63.8 - 67.0)	63.7 (60.6 - 66.7)	66.3 (63.3 - 69.3)
Guam	65.2 (63.6 - 66.9)	68.3 (66.7 - 69.9)	65.8 (62.5 - 69.1)	69.1 (65.9 - 72.4)
Guatemala	61.3 (59.7 - 63.0)	64.9 (63.3 - 66.5)	64.8 (61.4 - 68.2)	68.4 (65.1 - 71.7)
Guinea	52.7 (50.2 - 55.1)	55.1 (53.6 - 56.5)	55.1 (51.0 - 59.2)	58.8 (56.0 - 61.7)
Guinea-Bissau	52.7 (51.2 - 54.2)	56.0 (54.4 - 57.5)	57.6 (54.4 - 60.7)	60.0 (57.0 - 63.1)
Guyana	56.0 (54.5 - 57.5)	60.5 (59.0 - 62.0)	57.3 (54.6 - 60.0)	62.0 (59.1 - 64.9)
Honduras	62.5 (60.1 - 64.9)	63.0 (61.0 - 65.0)	63.9 (57.3 - 70.4)	63.7 (58.9 - 68.4)
Hungary	65.0 (63.4 - 66.6)	69.2 (67.6 - 70.8)	67.8 (64.7 - 70.8)	71.0 (68.2 - 73.9)
Iceland	71.8 (70.1 - 73.4)	73.4 (71.8 - 75.1)	73.3 (70.4 - 76.2)	75.2 (72.3 - 78.1)
India	60.8 (59.2 - 62.3)	60.7 (59.1 - 62.3)	64.2 (61.0 - 67.4)	64.5 (61.1 - 67.8)
Indonesia	61.9 (60.3 - 63.6)	63.9 (62.3 - 65.6)	64.3 (60.8 - 67.8)	66.5 (63.4 - 69.5)
Iran (Islamic Republic of)	66.6 (65.0 - 68.3)	67.4 (65.8 - 69.0)	70.3 (67.2 - 73.4)	70.3 (67.3 - 73.2)

Iraq	62.1 (60.1 - 64.1)	64.3 (62.5 - 66.1)	64.1 (59.3 - 68.9)	66.2 (62.0 - 70.4)
Ireland	70.3 (68.6 - 71.9)	70.9 (69.2 - 72.6)	72.5 (69.5 - 75.4)	72.4 (69.5 - 75.3)
Israel	71.9 (70.3 - 73.5)	72.1 (70.5 - 73.7)	73.4 (70.6 - 76.3)	73.6 (70.7 - 76.6)
Italy	70.8 (69.2 - 72.4)	72.2 (70.5 - 73.8)	73.0 (70.1 - 75.8)	73.7 (70.8 - 76.6)
Jamaica	65.7 (64.0 - 67.4)	67.1 (65.5 - 68.7)	65.7 (62.1 - 69.3)	67.3 (64.3 - 70.2)
Japan	72.7 (71.0 - 74.4)	75.3 (73.6 - 77.0)	74.4 (71.3 - 77.4)	76.8 (73.8 - 79.9)
Jordan	68.6 (67.0 - 70.2)	67.8 (66.1 - 69.5)	70.3 (67.2 - 73.4)	70.1 (66.7 - 73.5)
Kazakhstan	60.5 (58.8 - 62.3)	65.8 (64.2 - 67.5)	61.9 (58.1 - 65.7)	66.9 (63.5 - 70.2)
Kenya	56.7 (54.9 - 58.5)	59.7 (58.0 - 61.5)	57.7 (53.8 - 61.7)	61.6 (57.3 - 65.9)
Kiribati	51.8 (50.4 - 53.2)	55.8 (54.3 - 57.2)	53.7 (51.1 - 56.3)	57.7 (55.2 - 60.2)
Kuwait	69.8 (67.5 - 72.0)	71.8 (69.7 - 74.0)	71.3 (65.2 - 77.4)	74.2 (68.6 - 79.8)
Kyrgyzstan	62.9 (61.1 - 64.7)	66.8 (64.7 - 68.8)	65.1 (61.0 - 69.2)	68.3 (64.6 - 71.9)
Lao People's Democratic Republic	60.2 (58.6 - 61.7)	62.8 (61.2 - 64.3)	66.0 (63.0 - 68.9)	68.4 (65.4 - 71.5)
Latvia	63.8 (62.0 - 65.6)	69.2 (67.5 - 70.9)	66.4 (62.1 - 70.7)	71.0 (67.7 - 74.4)
Lebanon	65.0 (63.4 - 66.6)	67.4 (65.9 - 68.9)	66.9 (63.7 - 70.0)	69.1 (66.4 - 71.7)
Lesotho	42.6 (41.1 - 44.1)	46.7 (44.9 - 48.6)	40.4 (37.3 - 43.5)	44.1 (40.0 - 48.2)
Liberia	58.5 (55.6 - 61.3)	57.8 (55.5 - 60.1)	66.4 (56.7 - 76.0)	63.9 (57.2 - 70.6)
Libya	64.6 (62.9 - 66.3)	65.5 (63.9 - 67.0)	64.9 (61.2 - 68.6)	66.1 (62.9 - 69.3)
Lithuania	63.7 (62.0 - 65.4)	69.7 (68.0 - 71.3)	65.7 (61.8 - 69.6)	71.2 (68.1 - 74.3)
Luxembourg	71.0 (69.4 - 72.6)	71.6 (70.0 - 73.2)	73.7 (70.6 - 76.7)	73.1 (70.2 - 76.0)
Madagascar	57.7 (56.1 - 59.4)	58.3 (56.7 - 59.9)	61.2 (57.6 - 64.9)	61.4 (57.8 - 64.9)
Malawi	54.9 (53.0 - 56.9)	59.1 (57.0 - 61.1)	59.6 (54.3 - 64.8)	65.3 (59.4 - 71.2)
Malaysia	64.6 (63.0 - 66.3)	66.9 (65.3 - 68.6)	65.6 (62.5 - 68.6)	67.8 (64.6 - 71.0)
Maldives	69.4 (67.6 - 71.1)	70.0 (68.2 - 71.7)	73.3 (69.9 - 76.8)	74.9 (70.9 - 79.0)
Mali	54.8 (53.3 - 56.2)	54.8 (53.3 - 56.2)	58.9 (55.8 - 61.9)	59.1 (56.4 - 61.9)
Malta	70.6 (69.0 - 72.1)	71.8 (70.2 - 73.5)	72.3 (69.5 - 75.1)	73.3 (70.4 - 76.2)
Marshall Islands	56.8 (55.3 - 58.2)	58.0 (56.5 - 59.4)	57.8 (55.1 - 60.6)	58.0 (55.6 - 60.4)
Mauritania	62.9 (61.3 - 64.5)	61.9 (60.4 - 63.5)	66.8 (63.8 - 69.8)	65.4 (62.7 - 68.2)
Mauritius	63.5 (62.0 - 65.0)	67.1 (65.5 - 68.6)	65.0 (62.3 - 67.8)	68.2 (65.4 - 71.0)
Mexico	64.0 (62.4 - 65.5)	67.0 (65.4 - 68.6)	65.2 (62.3 - 68.1)	68.4 (65.4 - 71.4)
Micronesia (Federated States of)	55.3 (53.8 - 56.7)	58.3 (56.8 - 59.8)	56.2 (53.6 - 58.8)	59.0 (56.3 - 61.7)
Monaco	68.6 (67.1 - 70.1)	69.8 (68.2 - 71.5)	69.4 (66.8 - 71.9)	70.0 (67.3 - 72.7)
Mongolia	57.0 (55.4 - 58.7)	64.2 (62.5 - 65.9)	58.7 (55.2 - 62.2)	67.1 (63.4 - 70.8)
Montenegro	64.6 (63.0 - 66.2)	67.9 (66.4 - 69.5)	65.1 (62.2 - 67.9)	67.9 (65.2 - 70.7)
Morocco	63.4 (61.5 - 65.4)	64.1 (62.6 - 65.7)	65.1 (61.3 - 68.8)	66.1 (63.4 - 68.8)
Mozambique	47.8 (45.9 - 49.8)	52.8 (50.3 - 55.3)	49.6 (46.3 - 52.9)	55.4 (51.0 - 59.7)
Myanmar	59.4 (57.3 - 61.4)	63.2 (61.2 - 65.1)	64.4 (58.8 - 70.0)	67.8 (62.9 - 72.7)
Namibia	54.0 (52.3 - 55.7)	59.2 (57.2 - 61.2)	54.6 (50.6 - 58.5)	60.0 (54.7 - 65.2)
Nauru	54.2 (52.8 - 55.6)	57.8 (56.4 - 59.3)	54.4 (51.8 - 57.0)	58.0 (55.4 - 60.6)
Nepal	61.1 (59.3 - 62.8)	62.5 (60.8 - 64.2)	65.1 (61.6 - 68.5)	67.2 (63.7 - 70.6)
Netherlands	70.8 (69.1 - 72.4)	70.8 (69.2 - 72.4)	72.5 (69.6 - 75.4)	71.6 (68.7 - 74.4)
New Zealand	69.1 (67.6 - 70.7)	70.5 (68.8 - 72.1)	71.2 (68.3 - 74.1)	72.0 (69.1 - 74.8)
Nicaragua	63.9 (62.0 - 65.8)	67.3 (65.4 - 69.1)	65.2 (60.9 - 69.5)	67.9 (64.2 - 71.5)
Niger	55.6 (54.1 - 57.2)	56.3 (54.7 - 57.8)	61.4 (58.1 - 64.8)	62.0 (58.9 - 65.1)

Nigeria	55.8 (54.3 - 57.2)	56.8 (55.4 - 58.3)	59.2 (56.4 - 62.0)	60.0 (57.2 - 62.8)
Niue	60.0 (58.6 - 61.5)	62.9 (61.4 - 64.3)	60.7 (57.9 - 63.5)	63.2 (60.5 - 65.8)
North Macedonia	64.4 (62.8 - 65.9)	66.6 (64.9 - 68.2)	65.4 (62.4 - 68.4)	67.6 (64.6 - 70.5)
Northern Mariana Islands	62.3 (60.9 - 63.8)	65.8 (64.2 - 67.4)	62.5 (59.9 - 65.1)	66.8 (64.0 - 69.5)
Norway	70.8 (69.2 - 72.5)	71.3 (69.7 - 73.0)	73.0 (69.9 - 76.0)	72.5 (69.6 - 75.3)
Oman	64.8 (63.1 - 66.6)	65.0 (63.4 - 66.7)	67.0 (63.5 - 70.5)	66.7 (63.5 - 70.0)
Pakistan	57.4 (55.9 - 58.9)	57.1 (55.6 - 58.6)	58.7 (55.9 - 61.4)	58.6 (55.8 - 61.4)
Palau	57.8 (56.4 - 59.3)	61.4 (59.9 - 62.8)	58.5 (56.1 - 60.9)	61.9 (59.4 - 64.4)
Palestine	63.8 (62.1 - 65.5)	64.7 (63.1 - 66.4)	65.5 (61.3 - 69.7)	66.0 (62.9 - 69.2)
Panama	68.0 (66.4 - 69.6)	70.6 (68.9 - 72.2)	68.9 (65.9 - 71.8)	71.6 (68.6 - 74.5)
Papua New Guinea	55.7 (54.3 - 57.1)	57.1 (55.6 - 58.5)	56.4 (53.9 - 58.9)	57.5 (55.0 - 59.9)
Paraguay	64.9 (63.3 - 66.4)	67.7 (66.2 - 69.3)	64.8 (62.2 - 67.4)	68.2 (65.5 - 71.0)
Peru	70.0 (68.3 - 71.8)	70.8 (69.0 - 72.6)	73.9 (70.0 - 77.7)	74.0 (70.1 - 77.9)
Philippines	60.7 (59.1 - 62.2)	64.6 (63.0 - 66.1)	61.7 (58.8 - 64.7)	65.5 (62.8 - 68.3)
Poland	65.7 (64.2 - 67.3)	71.2 (69.6 - 72.8)	68.2 (65.3 - 71.1)	73.3 (70.4 - 76.2)
Portugal	69.5 (67.9 - 71.1)	71.5 (69.8 - 73.2)	72.1 (69.2 - 75.1)	73.4 (70.5 - 76.4)
Puerto Rico	67.8 (66.2 - 69.4)	71.7 (70.1 - 73.3)	69.9 (66.7 - 73.2)	73.1 (70.1 - 76.1)
Qatar	67.2 (65.3 - 69.1)	63.7 (62.0 - 65.4)	69.4 (64.8 - 74.0)	64.5 (61.2 - 67.7)
Republic of Korea	71.3 (69.7 - 73.0)	74.1 (72.4 - 75.8)	75.1 (72.1 - 78.1)	77.1 (74.1 - 80.1)
Republic of Moldova	62.5 (60.9 - 64.1)	67.9 (66.3 - 69.5)	64.3 (61.0 - 67.5)	69.9 (66.5 - 73.3)
Romania	64.1 (62.5 - 65.8)	69.1 (67.5 - 70.8)	66.0 (62.8 - 69.1)	71.0 (68.0 - 74.1)
Russian Federation	60.7 (58.9 - 62.5)	67.1 (65.5 - 68.7)	62.4 (58.1 - 66.8)	68.1 (65.0 - 71.3)
Saint Kitts and Nevis	61.1 (59.7 - 62.6)	65.8 (64.0 - 67.6)	62.3 (59.6 - 64.9)	68.3 (64.1 - 72.5)
Saint Lucia	63.4 (61.8 - 65.0)	66.9 (65.3 - 68.4)	64.4 (61.6 - 67.3)	68.4 (65.5 - 71.4)
Saint Vincent and the Grenadines	62.3 (60.7 - 63.8)	64.7 (63.1 - 66.3)	62.6 (59.9 - 65.4)	65.4 (62.6 - 68.3)
Samoa	61.7 (60.2 - 63.2)	62.1 (60.5 - 63.6)	63.2 (60.3 - 66.0)	62.4 (59.7 - 65.0)
San Marino	70.5 (68.8 - 72.1)	71.6 (70.0 - 73.2)	71.3 (68.5 - 74.2)	72.0 (69.3 - 74.7)
Sao Tome and Principe	62.0 (60.5 - 63.5)	63.1 (61.6 - 64.6)	64.4 (61.6 - 67.2)	65.4 (62.5 - 68.4)
Saudi Arabia	64.4 (62.9 - 65.8)	65.2 (63.6 - 66.8)	65.9 (63.2 - 68.6)	67.1 (64.3 - 69.8)
Senegal	59.7 (58.3 - 61.2)	60.8 (59.4 - 62.3)	63.3 (60.6 - 66.0)	64.0 (61.3 - 66.6)
Serbia	65.1 (63.5 - 66.8)	67.9 (66.3 - 69.5)	66.6 (63.3 - 69.8)	69.2 (66.1 - 72.4)
Seychelles	62.5 (60.9 - 64.0)	66.8 (65.2 - 68.3)	63.5 (60.7 - 66.4)	67.1 (64.4 - 69.8)
Sierra Leone	54.2 (52.6 - 55.9)	54.7 (53.0 - 56.4)	58.6 (54.5 - 62.8)	58.0 (53.7 - 62.3)
Singapore	74.1 (72.4 - 75.8)	75.6 (73.9 - 77.2)	77.4 (74.3 - 80.5)	78.2 (75.2 - 81.2)
Slovakia	65.6 (64.0 - 67.2)	70.0 (68.5 - 71.6)	68.0 (65.1 - 70.8)	71.6 (68.7 - 74.4)
Slovenia	68.6 (67.0 - 70.3)	72.6 (71.0 - 74.2)	71.2 (68.0 - 74.3)	74.6 (71.8 - 77.4)
Solomon Islands	52.0 (50.7 - 53.3)	53.6 (52.3 - 55.0)	53.0 (50.6 - 55.3)	54.5 (52.1 - 56.8)
Somalia	50.3 (48.8 - 51.8)	53.4 (52.0 - 54.8)	53.4 (50.1 - 56.6)	56.5 (53.8 - 59.3)
South Africa	53.8 (52.0 - 55.7)	57.3 (55.3 - 59.3)	54.0 (49.7 - 58.2)	57.0 (52.0 - 61.9)
South Sudan	53.9 (52.5 - 55.3)	55.9 (54.5 - 57.4)	56.8 (54.0 - 59.7)	58.7 (56.1 - 61.3)
Spain	71.2 (69.6 - 72.8)	72.5 (70.8 - 74.2)	73.5 (70.6 - 76.4)	73.9 (70.9 - 76.9)
Sri Lanka	65.5 (63.5 - 67.4)	69.2 (67.4 - 70.9)	68.1 (63.3 - 72.9)	71.1 (67.2 - 74.9)
Sudan	61.4 (59.9 - 62.9)	62.2 (60.7 - 63.8)	65.4 (62.5 - 68.3)	65.9 (63.1 - 68.7)
Suriname	61.0 (59.3 - 62.7)	64.3 (62.7 - 65.9)	61.6 (58.2 - 65.1)	65.2 (62.0 - 68.5)

Sweden	71.5 (69.9 - 73.2)	71.6 (70.0 - 73.2)	73.2 (70.4 - 76.1)	72.6 (69.8 - 75.3)
Switzerland	72.0 (70.4 - 73.6)	72.4 (70.7 - 74.0)	74.5 (71.6 - 77.5)	73.7 (70.9 - 76.5)
Syrian Arab Republic	62.8 (59.4 - 66.1)	63.8 (61.6 - 65.9)	64.1 (54.1 - 74.0)	64.8 (59.1 - 70.4)
Taiwan (Province of China)	69.3 (67.7 - 70.9)	72.4 (70.7 - 74.1)	70.8 (67.9 - 73.6)	74.3 (71.3 - 77.3)
Tajikistan	60.5 (58.3 - 62.7)	62.6 (60.8 - 64.4)	62.1 (56.1 - 68.1)	63.5 (59.9 - 67.1)
Thailand	66.3 (64.7 - 68.0)	71.1 (69.4 - 72.7)	68.7 (65.3 - 72.1)	73.3 (70.4 - 76.3)
Timor-Leste	60.9 (57.6 - 64.2)	63.1 (61.2 - 65.0)	64.1 (53.9 - 74.3)	66.8 (62.2 - 71.4)
Togo	55.2 (53.5 - 56.8)	58.8 (57.3 - 60.3)	57.0 (53.7 - 60.2)	61.5 (58.5 - 64.4)
Tokelau	65.1 (63.5 - 66.6)	61.7 (60.3 - 63.2)	66.2 (63.5 - 68.9)	62.8 (60.2 - 65.5)
Tonga	62.2 (60.5 - 63.8)	65.6 (64.0 - 67.1)	62.4 (59.3 - 65.5)	66.2 (63.3 - 69.2)
Trinidad and Tobago	63.5 (61.9 - 65.1)	67.0 (65.5 - 68.6)	64.8 (61.8 - 67.7)	68.6 (65.8 - 71.5)
Tunisia	66.9 (65.4 - 68.4)	68.7 (67.1 - 70.3)	68.2 (65.4 - 71.1)	70.2 (67.4 - 73.0)
Turkey	67.6 (65.8 - 69.3)	68.4 (66.6 - 70.1)	70.7 (66.8 - 74.6)	70.7 (67.0 - 74.4)
Turkmenistan	60.4 (58.7 - 62.1)	65.1 (63.3 - 66.8)	61.8 (58.0 - 65.5)	66.6 (63.1 - 70.1)
Tuvalu	59.4 (57.8 - 61.0)	60.9 (59.0 - 62.8)	61.6 (58.1 - 65.2)	63.1 (58.2 - 67.9)
Uganda	56.1 (54.5 - 57.8)	60.8 (59.1 - 62.4)	62.4 (58.5 - 66.3)	67.8 (63.8 - 71.8)
Ukraine	61.5 (59.9 - 63.1)	68.0 (66.4 - 69.6)	62.7 (59.2 - 66.2)	69.2 (66.2 - 72.3)
United Arab Emirates	64.3 (62.7 - 65.8)	65.1 (63.4 - 66.9)	65.6 (62.7 - 68.4)	66.6 (62.8 - 70.4)
United Kingdom	68.8 (67.2 - 70.4)	69.7 (68.1 - 71.3)	70.5 (67.6 - 73.3)	70.9 (68.0 - 73.7)
United Republic of Tanzania	58.3 (56.6 - 60.0)	60.1 (58.5 - 61.7)	62.7 (58.8 - 66.5)	64.6 (60.8 - 68.5)
United States of America	64.7 (63.1 - 66.3)	66.6 (65.0 - 68.2)	65.6 (62.8 - 68.4)	66.8 (64.2 - 69.5)
United States Virgin Islands	62.1 (60.6 - 63.6)	68.6 (67.0 - 70.2)	62.1 (59.5 - 64.7)	69.4 (66.6 - 72.3)
Uruguay	65.6 (64.1 - 67.2)	69.4 (67.8 - 71.0)	66.9 (64.2 - 69.7)	70.4 (67.6 - 73.3)
Uzbekistan	58.8 (57.1 - 60.6)	61.5 (59.9 - 63.2)	58.8 (55.7 - 61.9)	61.2 (58.4 - 64.1)
Vanuatu	56.4 (55.0 - 57.7)	59.1 (57.7 - 60.6)	57.1 (54.6 - 59.7)	59.6 (57.0 - 62.2)
Venezuela (Bolivarian Republic of)	63.2 (61.5 - 64.8)	67.7 (66.1 - 69.4)	63.7 (60.2 - 67.2)	68.8 (65.5 - 72.0)
Viet Nam	63.1 (61.5 - 64.6)	68.9 (67.3 - 70.5)	64.6 (61.9 - 67.3)	70.8 (67.9 - 73.6)
Yemen	58.3 (56.4 - 60.3)	59.3 (57.1 - 61.4)	61.1 (56.0 - 66.2)	62.0 (55.9 - 68.0)
Zambia	53.5 (51.6 - 55.4)	57.5 (55.4 - 59.5)	56.7 (51.5 - 61.9)	62.0 (56.3 - 67.6)
Zimbabwe	51.4 (49.5 - 53.4)	54.9 (52.7 - 57.2)	51.0 (45.9 - 56.0)	54.6 (48.9 - 60.4)

‡ The measure of the values in this table is in years, and the values in brackets represent the predicted 95% confidence interval (CI).
